# Fibrous Polymeric Composites Based on Alginate Fibres and Fibres Made of Poly-ε-caprolactone and Dibutyryl Chitin for Use in Regenerative Medicine

**DOI:** 10.3390/molecules18033118

**Published:** 2013-03-08

**Authors:** Maciej Boguń, Izabella Krucińska, Agnieszka Kommisarczyk, Teresa Mikołajczyk, Marta Błażewicz, Ewa Stodolak-Zych, Elżbieta Menaszek, Anna Ścisłowska-Czarnecka

**Affiliations:** 1 Department of Material and Commodity Sciences and Textile Metrology, Technical University of Lodz, ul. Żeromskiego 116, 90-924 Łódź, Poland; 2 Department of Biomaterials, Faculty of Materials Science and Ceramics, AGH University of Science and Technology, al. Mickiewicza 30, 30-059 Krakow, Poland; 3 Department of Cytology, Faculty of Pharmacy, CMUJ — Jagiellonian University Medical Collage, ul. Medyczna 9, 30-688 Krakow, Poland; 4 Department of Physiotherapy, Faculty of Rehabilitation, University School Of Physical Education, Al. Jana Pawla II 78, 31-571 Krakow, Poland

**Keywords:** dibutyryl chitin, poly-ε-caprolactone, alginate fibres, biodegradable polymers

## Abstract

This work concerns the production of fibrous composite materials based on biodegradable polymers such as alginate, dibutyryl chitin (DBC) and poly-ε-caprolactone (PCL). For the production of fibres from these polymers, various spinning methods were used in order to obtain composite materials of different composition and structure. In the case of alginate fibres containing the nanoadditive tricalcium phosphate (TCP), the traditional method of forming fibres wet from solution was used. However in the case of the other two polymers the electrospinning method was used. Two model systems were tested for biocompatibility. The physicochemical and basic biological tests carried out show that the submicron fibres produced using PCL and DBC have good biocompatibility. The proposed hybrid systems composed of micrometric fibres (zinc and calcium alginates containing TCP) and submicron fibres (DBC and PCL) meet the requirements of regenerative medicine. The biomimetic fibre system, the presence of TCP nanoadditive, and the use of polymers with different resorption times provide a framework with specific properties on which bone cells are able to settle and proliferate.

## 1. Introduction

The needs of the market for modern implant materials are stimulating the rapid development of regenerative medicine, and especially of tissue engineering, whose task is to assist the reconstruction of damaged tissues [1]. The development of this field of medicine based on a new class of biomaterial engineering solutions is making a significant contribution in terms of both shortening patient hospitalization times and bringing notable economic benefits.

The use of polymeric composites in the treatment of bone tissue defects has the aim of reproducing as closely as possible the damaged tissues of the living body. A particularly important role among materials in this category is played by composites made from biodegradable and bioresorbable polymers. The natural biodegradable polymers most often used at present in medical applications include various types of alginates and chitin derivatives [2]. 

**Figure 1:**
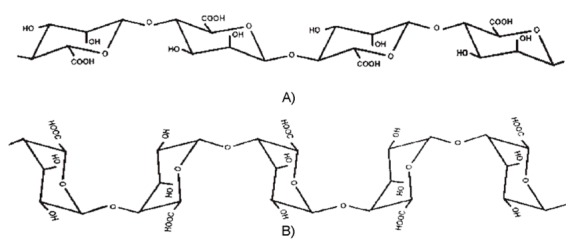
Chemical structure of macromolecules of polymannuronic acid (**A**) and polyguluronic acid (**B**); based on [4].

Alginates are linear polysaccharides built of radicals of β-d-mannuronic acid (M) and α-l-guluronic acid (G) [3,4], which fact has a significant influence on the properties of the fibres obtained from this polymer. Figure 1 shows diagrams of the macromolecules of polymannuronic and polyguluronic acids.

The properties of this polymer of the greatest importance from a medical point of view include particularly its non-toxicity and its antibacterial action. Alginates are used in tissue engineering for the treatment and regeneration of skin, cartilaginous and bone tissue, the liver and tissues of the heart muscle [5,6,7,8,9].

**Figure 2:**
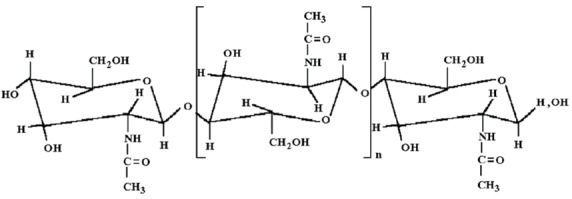
Chemical structure of chitin.

Another natural polymer which is coming to be used increasingly often for medical applications is chitin and its derivatives. Like alginate, chitin is a linear polysaccharide; its chemical structure is shown in Figure 2.

This substance is built up of units of N-acetylglucosamine joined with β-(1-4) glycoside bonds. It is extracted from many organisms, including shrimps, krill, crabs, lobsters and various types of insects [10]. The polymer has very good biological properties, namely the ability to regenerate skin, cartilaginous and bone tissue [11,12]. However one of its drawbacks is its poor solubility and the presence of contaminants in the form of such compounds as proteins or various inorganic compounds. The problem of the solubility of this polymer was solved as a result of work done on the improvement of chitin solubility at the University of Technology in Lódź, Poland. This work led to the obtaining of a derivative of chitin called dibutyryl chitin (DBC) [13]. Unlike chitin itself, dibutyryl chitin dissolves well in many commonly used solvents such as acetone and ethyl alcohol. 

The synthetic biodegradable and bioresorbable polymers widely used in medicine include polylactide, polyglycolide, poly-ε-caprolactone and their copolymers. Polycaprolactone is one of a group of biodegradable aliphatic polyesters with a linear, semi-crystalline structure. Its great advantage over other polymers of this group is its relatively low price, which has led to its becoming widely used in medical materials. It shows good solubility, a low melting point of 60 °C, and exceptionally good ability to mix with many other polymers. It is used in medicine in the form of surgical threads, in controlled drug release systems, in dentistry and in vascular surgery [14,15,16,17].

At the same time, a factor of great importance in modelling the properties of obtained composite materials is the use of nanotechnology. Through the introduction of additives of nanometric dimensions into the polymeric matrix, or the use of electrospinning techniques, it is possible to improve significantly the properties of many synthetic materials relative to materials obtained using traditional methods. Hence by using nanotechnology it is possible, for example, to shape the properties of composites with regard to their medical applications, improving their biocompatibility, biodegradability, or osteoconductive and osteoinductive properties [18]. 

The goal of the present work was to develop and analyse the properties of a new type of fibrous composite materials obtained based on the following types of fibres:

- alginate fibres containing TCP nanoadditive (ZA).
- alginate fibres containing TCP nanoadditive (CA).
- made from PCL and DBC using the electrospinning method.

The composites obtained are intended for use in tissue engineering, to treat bone tissue defects. 

## 2. Results and Discussion

### 2.1. Properties of Alginate Fibres

**Figure 3:**
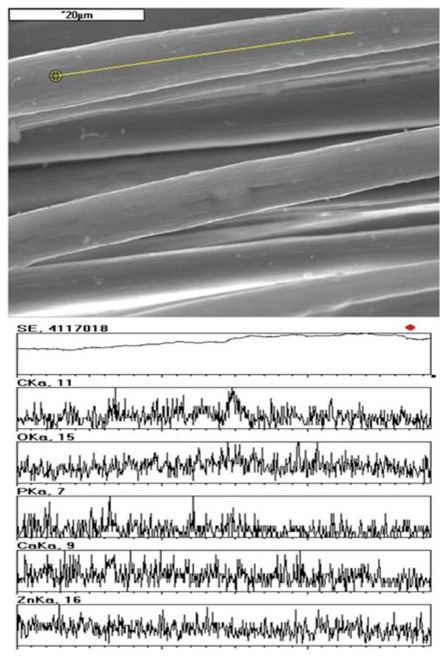
SEM+EDS analysis of zinc alginate fibres containing TCP.

The study used zinc and calcium alginate fibres with 3% TCP nanoadditive introduced into the fibre material. The conditions of the forming process and the fibre properties were optimized with respect to the intended use of the fibres, as described in our earlier publications [19,20,21,22]. Table 1 gives the basic properties of the alginate fibres used. SEM + EDS morphological analysis also showed the presence of characteristic elements originating from the nanoadditive. An example of this, for zinc alginate fibres, is shown in Figure 3.

**Table 1:** Properties of the alginate fibres used to produce composites [19,20,21,22].

Type of fibres	As-spun draw ratio [%]	Total draw ratio [%]	Total pores volume, [cm^3^/g]	Tenacity [cN/tex]	Elongation at break [%]
Zinc alginate with TCP	+70	224.20	0.31	23.99 ± 0.80	5.57 ± 0.48
Calcium alginate with TCP	+70	89.27	0.38	24.39 ± 0.71	10.39 ± 0.41

The zinc alginate fibres obtained wet from solution and used in the production of *variant I* fibrous composite were processed into nonwoven fabric using a carding device (BEFAMA laboratory carding machine), and then subjected to a needling process. The parameters of this process and the properties of the fabrics obtained are presented in Table 2. In accordance with the concept for *variant II* composites, the alginate fibres were introduced in the form of segmental fibres (2–3 mm) into a PCL or DBC nonwoven fabric in the course of an electrospinning process.

**Table 2:** Conditions for the production of nonwoven fabric from alginate fibres and properties of the fabric.

Type of fabric	Needle type	Number of needling operations [1/cm2]	Depth of needling [mm]	Area density [g/m^2^]	Fabric thickness [mm]	Pore area [m^2^/g]	Average pore size [µm]
Zinc alginate fibres with TCP	15 × 18 × 40 × 3.5 RB	30	12	56	1.36	0.353	74.8

### 2.2. Properties of Fibrous Nonwoven Fabrics Obtained by Electrospinning

Based on preliminary tests of the electrospinning process, optimum conditions were selected for the production of nonwoven fabrics from dibutyryl chitin (DBC) and poly-ε-caprolactone (PCL), as shown in Table 3. 

**Table 3:** Optimum conditions for formation of nonwoven fabrics from DBC and PCL fibres where: Cp = % concentration by weight; T = ambient temperature; Φ = air relative humidity; U = supply voltage; h = distance from capillary to collector; ω = angular speed of collecting drum; P = angular speed of supply pump.

Type of polymer	Type of solvent	Cp [%]	T [°C]	Φ [%]	U [kV]	h [cm]	ω [1/min]	P [obr/min]
PCL	dichloromethane	7.5	13.6	48	35	50	20	1.1
DBC	ethyl alcohol	6.0	23.0	48	28	15	20	5.5

**Figure 4:**
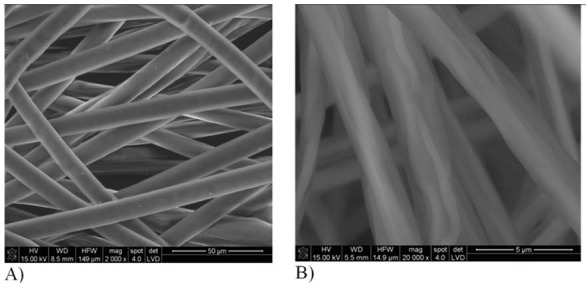
Morphology of fibres obtained by electrospinning. Fibres made from polycaprolactone (**A**) and dibutyryl chitin (**B**).

Morphological tests of PCL and DBC fibres obtained by electrospinning show that the PCL fibres have a diameter of around 11–15 μm, and their surface is smooth and uniform (Figure 4A). For DBC fibres the diameter was in the range 3–8 μm (Figure 4B), and the surface was much more irregular and creased, with cracks and scratches present.

Physicochemical tests show that the PCL fabric has a hydrophobic surface, with a wetting angle of 131 degrees. For the DBC fabric the wetting angle is 98 degrees. For the submicron fabrics tests were also performed on the short-term and long-term biodegradation process, as presented in partial form in [23]. In order to determine the character of the surface of the fabrics, their surface roughness was tested. The average roughness profile (R_a_) of the fabric measured in its central and extreme parts showed significant differences, while the parameters R_t_ and R_z_ differed only slightly (Table 4). This was probably a consequence of the differing thickness of the fabrics obtained by electrospinning. During these tests it was observed that the PCL fabric is a more compact material and adheres better to the base than the DBC fabric, which shows a tendency to become electrified. 

Wettability and roughness research enabled to determine the synergistic impact of those physicochemical properties. Indeed optimal water contact angle for materials used as bone regeneration biomaterials is about 50–70°. However osteoblasts are cells which respond to a large extent not only on the optimum hydrophilicity but above all, the scope of the substrate roughness, its orientation or in case of fibrous products fiber diameter [24]. Electrospinning method, used in our research, provide a roughness at submikron level (Ra about 1 µm), impacting on water contact angle value (hysteresis roughness) [25]. Traditionally processed PCL, for example by molding method, is characterized by water contact angle about 70°. Obtaining PCL in fibrous form of submicron and nanometric diameter increases roughness and decrease wettability of PCL (or DBC) nonwoven which is reflected into an increase in theta value (increase in our study PCL fibres to value 131°). What is more, diameter of PCL fibres 10–15 µm and DBC fibres 3–8 µm has an additional effect. DBC fibres which have smaller diametre are better wettable. Furthermore incubation of DBC fibres affect on stronger swelling what could impede adhesion and proliferation bone cells. PCL fibres are difficult to wettable and difficult to swell what significantly impedes osteoblasts subsidence. If nonwovens from those materials obtained by electrospinning cover with micrometric diameter alginate calcium/zinc the biopolymer layer (ZA or CA) will guaranty better hydration which enables wettability and osteoblasts subsidence on designed composite materials (PCL/ZA:CA theta = 78°, DBC/ZA:CA theta = 58°) (Table 4). 

**Table 4:** Physicochemical properties of submicron nonwoven fabrics obtained by electrospinning.

Type of fabric	Wetting angle [°]	Surface roughness in central part			Surface roughness in extreme part		
		R_a_ [µm]	R_t_ [µm]	R_z_ [µm]	R_a_ [µm]	R_t_ [µm]	R_z_ [µm]
PCL	131 ± 2.24	1.08 ± 0.12	0.83 ± 0.02	0.64 ± 0.06	1.84 ± 0.45	1.31 ± 0.29	1.46 ± 0.81
DBC	98 ± 1.78	0.96 ± 0.16	0.98 ± 0.09	0.54 ± 0.08	0.61 ± 0.02	0.87 ± 0.03	0.64 ± 0.12

For both types of fabrics obtained by electrospinning, an initial assessment of their bioactivity was made by incubating them in a solution of simulated body fluid (SBF). However in neither case was evidence of bioactivity of the fabrics observed, as incubation in SBF did not cause the crystallization of apatite structures. In addition, SEM + EDS analysis did not reveal the presence of the characteristic elements contained in apatite.

**Figure 5:**
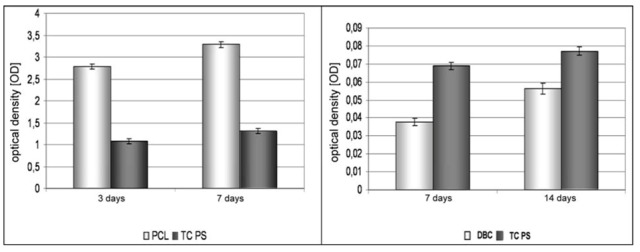
Data showing survival MTT activity cells contacted with the surface of PCL fabric (7-day culture) and DBC fabric (14-day culture).

An assessment was also made of the biocompatibility of the submicron nonwoven fabrics obtained by electrospinning, using biological tests with lines of MG-63 osteoblastoid cells. A mitochondrial activity test (MTT) was also performed, and the tendency of cells to adhere to the surface of the fabrics was analysed microscopically. In the case of PCL fabric a positive result was obtained after only 7 days, while the DBC fabrics confirmed their biocompatibility only on the 14th day of the experiment. The data presented in Figure 5 show that the PCL fabrics display high cell survival compared with the DBC fabrics. The lower cell survival in the case of DBC fabric is probably linked to the less hydrophobic nature of the fibres obtained from a chitin derivative, which may have a negative influence on the process of settlement of osteoblasts on the surface of such fibres.

### 2.3. Properties of Variant I Composites

**Figure 6:**
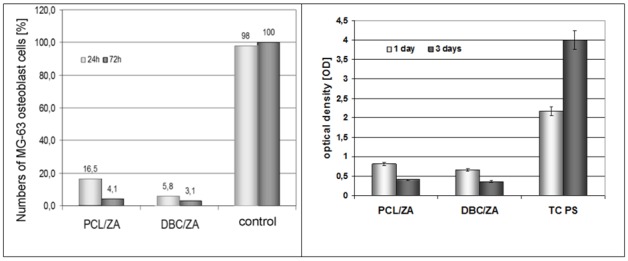
Data showing cell numbers and viability on the surface of composites containing zin alginate fibres.

**Figure 7:**
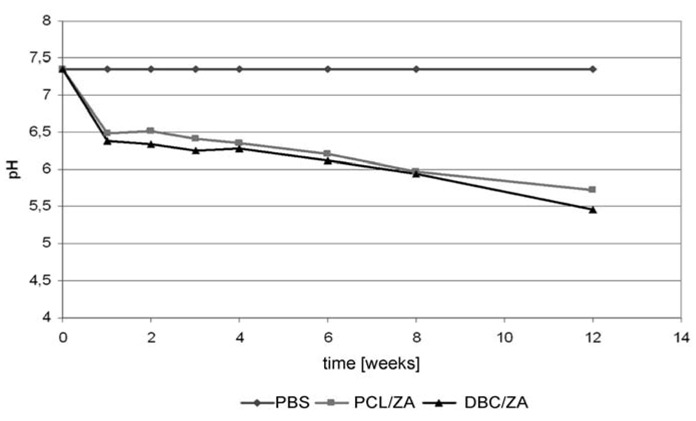
Change in pH of the medium for PCL/ZA and DBC/ZA composites in PBS.

With regard to the positive results for the biocompatibility of submicron structures, tests were performed on the proposed model composites containing zinc alginate fibres containing TCP (ZA). The numbers of cells in contact with these fibrous materials, in both cases—for the ZA/PCL system and for ZA/DBC—were found to have a tendency to decrease. A high cell death rate was observed, in comparison with the high level of viability on the control material (TC PS, Figure 6). There was also a change in the colour of the immersion medium, caused by a change of pH in the incubation fluid (Figure 7), which led to accelerated cell death. This was probably due to too great a concentration of zinc ions in the culture medium. Also, in the case of both types of materials, the unfavourable phenomenon of composite delamination was observed, while the zinc alginate fibres transformed into gel at a fairly fast rate. 

### 2.4. Properties of Variant II Composites

**Figure 8:**
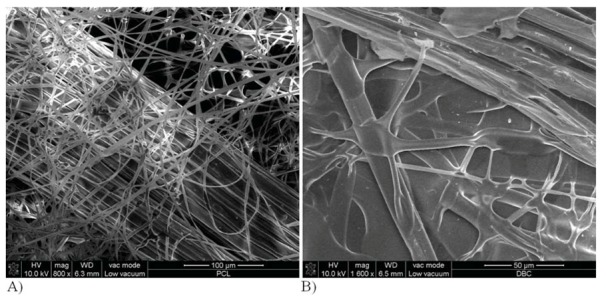
SEM image of fibrous composite: (**a**) from PCL; (**b**) from DBC.

In the formation of variant II composites, the quantity of zinc alginate fibres was reduced in favour of calcium alginate fibres. Zinc and calcium alginate fibres with TCP nanoadditive were mixed in a ratio of 5:95, and this mixture was introduced onto the PCL or DBC nonwoven fabric during the electrospinning process. Thus fibres of micrometric dimensions were placed between layers of submicron fibres. Figure 8 shows photographs of this composite taken using a scanning electron microscope (SEM).

In the case of this variant of the composite, the following model systems were used:

1. nonwoven fabric (electrospinning) with the addition of micrometric fibres in a ZA:CA ratio of 5:95, proportion of micro fibres 20%.
2. nonwoven fabric (electrospinning) with the addition of micrometric fibres in a ZA:CA ratio of 5:95, proportion of micro fibres 28%.

A smaller proportion of micrometric fibres was used in the case of the composite made from DBC because of the disturbance of the electrospinning process that occurred in the case of this polymer. This phenomenon was not observed when submicron fibres were obtained from poly-ε-caprolactone. 

Physicochemical tests showed that these materials have differing wettability depending on the system used (Table 5). Also the introduction of micrometric fibres (alginate fibres) into a fibrous matrix made of submicron fibres (PCL or DBC) ensures that differentiated roughness will be obtained (Table 5). A system in which the modifying phase with a tendency to degrade faster constitutes the smaller part of the composition (20–28%) enables the compatibility of the material with bone cells to be presumably better. Moreover the short alginate fibres may serve as an additional porogen in living body conditions. 

**Table 5:** Physicochemical properties of fibrous composites.

Type of fabric	pH of immersion medium		Wetting angle [°]	Surface roughness		
	water	PBS		R_a_ [µm]	R_t_ [µm]	R_z_ [µm]
PCL/ZA:CA	6.58	7.38	78.4 ± 2.3	8.85 ± 0.26	61.39 ± 1.84	47.12 ± 1.31
DBC/ZA:CA	6.59	7.39	58.2 ± 1.4	3.47 ± 0.14	46.65 ± 0.86	20.18 ± 0.62

**Figure 9:**
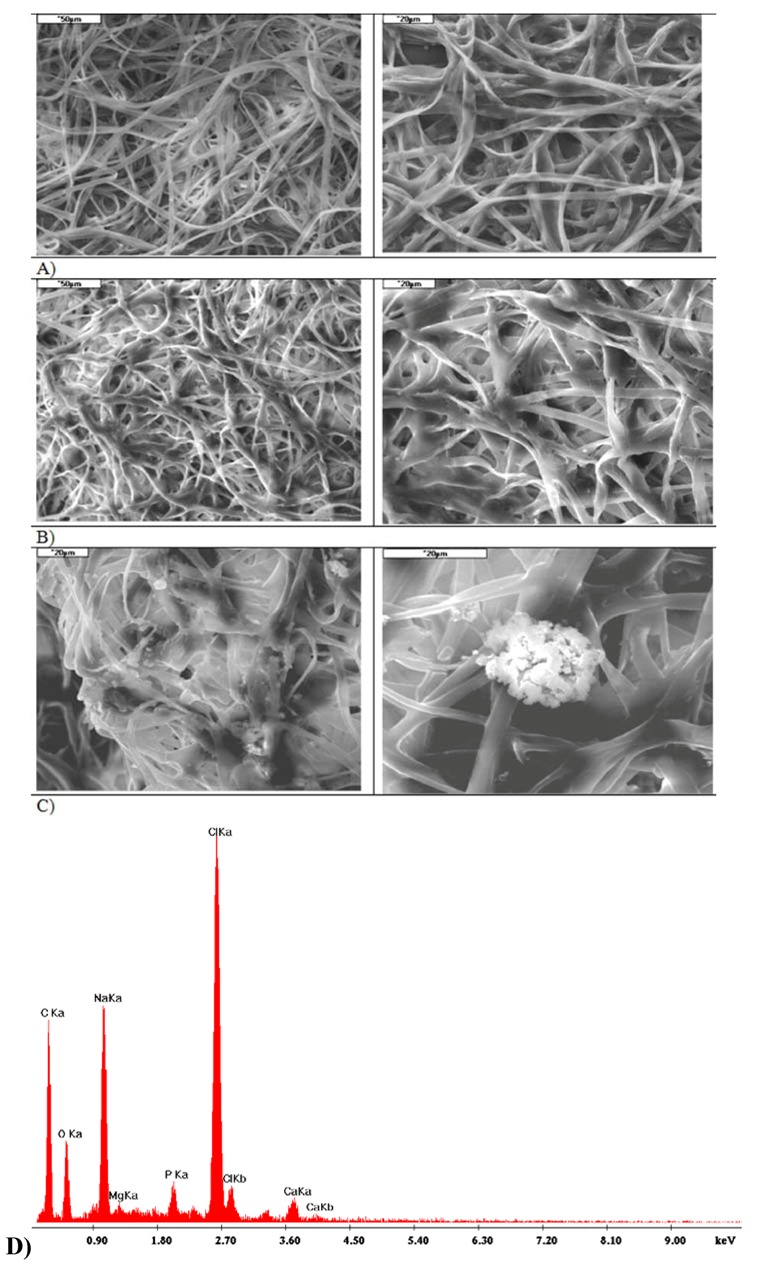
Microstructure and EDS of DBC/ZA:CA composite before and after incubation in PBS and SBF: (**A**) before incubation, (**B**) after incubation in PBS, (**C**) after incubation in SBF, (**D**) EDS analysis after incubation in SBF.

**Figure 10:**
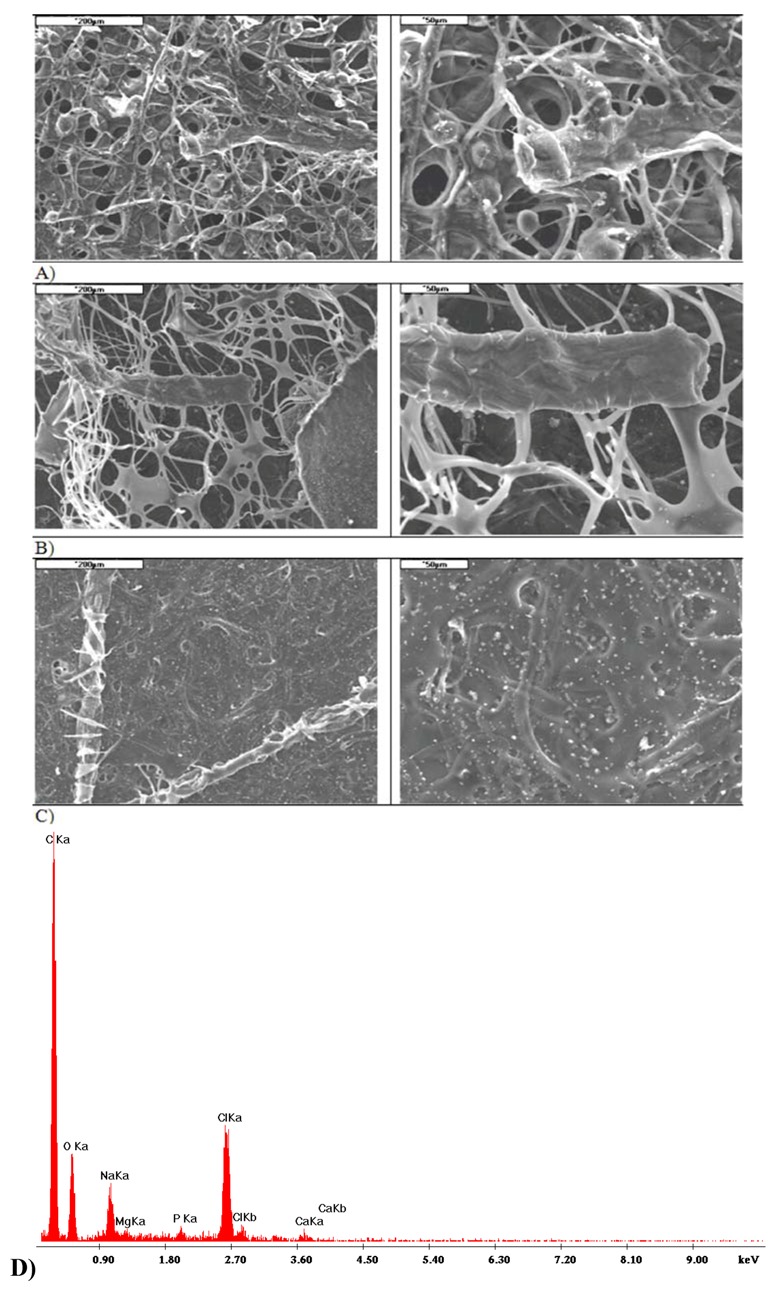
Microstructure of PCL/ZA:CA composite before and after incubation in PBS and SBF: (**A**) before incubation, (**B**) after incubation in PBS, (**C**) after incubation in SBF, (**D**) EDS analysis after incubation in SBF.

Accelerated degradation tests (temp. 80 °C) show that the DBC/ZA:CA and PCL/ZA:CA composites have a similar absorbability of approximately 480% (in 72 h). However in normal conditions (temp. 37 °C) the absorbability of DBC/ZA:CA is around 400%, while that of PCL/ZA:CA is around 250%. Macroscopic observations of the fabrics after short-term incubation did not reveal any macroscopic changes. However as a result of SEM microscopic observations changes in microstructure were noticed, resulting from the initiated degradation process. In addition a bioactivity test confirmed that nucleation of apatite may have occurred in some places (Figure 9), since EDS analysis revealed small amounts of phosphorus on DBC/ZA:CA fabrics incubated for 7 days in simulated plasma (SBF). The clusters of apatite that appear in this case (Figure 9) do not take their traditional cauliflower-like forms, this probably being connected with the tendency of those materials to swell. In the case of PCL/ZA:CA fabrics the faster-degrading alginate fibres cause the development of additional porous structure, and the alginate is deposited on the surface of the PCL fibres. Also for composites of this type after a period of 14 days’ incubation the formation of scattered apatite structures becomes visible. Hence in this case too we are dealing with a bioactive fibrous composite (Figure 10). The pH values for the immersion medium—water and a phosphate buffer—given in Table 4 do not indicate significant changes in the value of this parameter when different types of submicron layers are used. For both types of fabric the pH of the immersion medium was at a safe level, allowing these fabrics to be used as implant materials.

**Figure 11:**
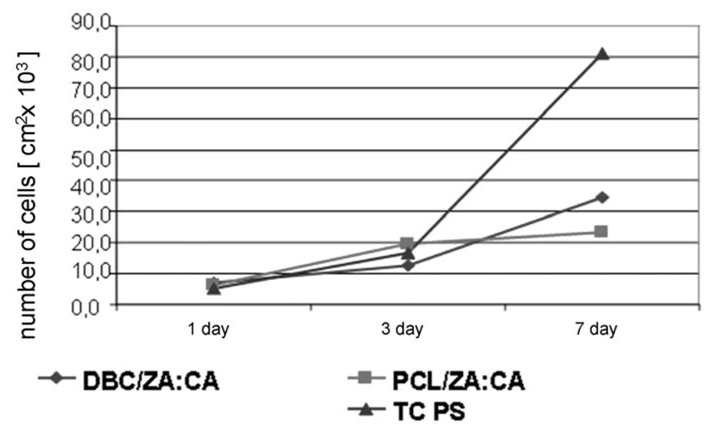
Graph of number of cells as a function of time, for composite materials and the control sample.

As in the previous case, biological tests were performed using lines of MG-63 osteoblastoid cells. A week-long culture of cells brought into contact with the fabric surfaces showed that the best properties stimulating the material towards faster proliferation were possessed by the DBC/ZA:CA fabric, since it was in this case that the fastest growth in the number of cells was observed after 7 days’ culture (Figure 11). The lower viability of cells on the PCL/ZA:CA fabric may indicate that the culture time was too short. That fabric demonstrated lower absorbability and tendency to create a domain surface after 7 days’ incubation. Such a form was probably more favourable to the osteoblast adhesion process. 

### 2.5. Comparative Analysis of I and II Variant Composites

The tests carried out on submicron fibres obtained by electrospinning showed that they are a biocompatible material characterized by good adhesion of cells to their surface. At the same time, degradation in an artificial biological environment is significantly faster for the DBC material compared with degradation of PCL fibres. Biological tests performed on PCL nonwoven fabric show an improvement in the ability of osteoblastoid cells to adhere to the fibrous base compared with the TCPS control. This supports the conclusion that it will be possible to stimulate the proliferation of bone cells to a material of this type. In the case of the DBC fabric the bone cell viability test shows lower survival of MG-63 line cells in contact with the material than in the case of TCPS. Nonetheless there is a visible rise in the survival of osteoblasts in a set culture time, which supports the conclusion of an improvement in adhesion and the possibility of proliferation of cells on these materials (Figure 11).

**Figure 12:**
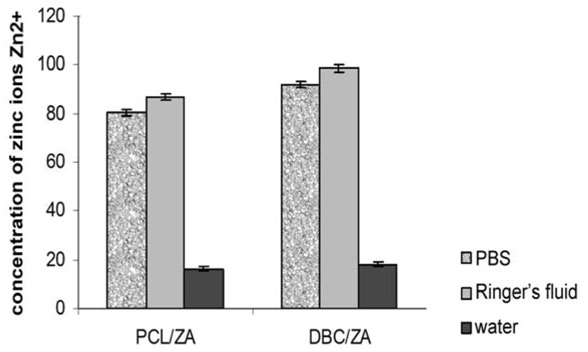
Changes in concentration of zinc ions in the immersion media PBS, Ringer’s fluid and water after 7 days’ incubation in conditions of short-term degradation (80 °C/5% CO_2_).

The model systems obtained under variant I displayed unfavourable properties. During their incubation in culture media, fairly rapid degradation of the zinc alginate fibrous phase took place, with delamination of the composite materials. The tests of bone cell survival returned fairly low values of that parameter, which may indicate a negative cellular response. In the case of both PCL and DBC composite systems containing zinc alginate fibres there was a change in the colour of the immersion medium, caused by a reduction in pH to around 5.5. Tests to identify the elements passing into the immersion medium (Figure 12) confirmed that the first stage of degradation of the material involves the micrometric zinc alginate (ZA) fibres. In this case partial hydrolysis of the zinc alginate may occur, with larger quantities being released into the culture medium.

However neither the low results for survival of cells in contact with composites with high zinc alginate fibre content, nor the delamination of this composite in the culture medium, disqualify this material from use in implants for the regeneration of tissue in the form of fillers. They lead to the assumption, though, that the proportion of zinc alginate fibres should be limited. It is not appropriate to eliminate zinc alginate completely in this case, because of the role played by zinc in the metabolism of tissues — it is present in the active centres of many enzymes, and also appears during bone mineralization processes. Moreover its rapid degradation creates possibilities for using these fibres as a carrier for a bioactive substance, in this case the nanoadditive TCP.

The composites obtained under variant II showed significantly better properties than the previously discussed implant material. In the case of these composites the phenomenon of delamination of implant materials was not observed. Also the results for survival of contacting MG-63 cells were much higher. The observed bioactivity of these materials creates much wider possibilities for their use in regenerative medicine. Reducing the proportion of zinc alginate fibres in favour of calcium alginate fibres made it possible to obtain more stable systems, for which no changes in the pH of the culture medium occurred. Moreover the short alginate fibres used act as a reservoir of bactericidal ions (Zn^2+^) as well as bioactive ones (Ca^2+^). They may also serve as carriers for a bioactive substance. However the use of two polymers with different wettability—hydrophobic (PCL or DBC) and hydrophilic (alginate fibres)—enables covering *in situ* with a layer of different physicochemical character, which is favourable from the point of view of the applications of these systems. 

## 3. Experimental

### 3.1. Raw Materials

The sodium alginate used in the study was produced by FMC Biopolymer (Haugesund, Norway) under the trade name Protanal LF 10/60LS, with intrinsic viscosity η = 3.16 dL/g. Its degree of polydispersity was 6.8, determined by gel chromatography (SEC/GPC). The nanoadditive used in the preparation of alginate fibres was suitably ground β-tricalcium phosphate (TCP) from Chempur (Piekary Śląskie, Poland). The nanoadditive was introduced into the fibre material at the stage of preparation of the spinning solution, and its average particle size range was 80–110 nm. The poly-ε-caprolactone (PCL) used was a commercial product of Sigma Aldrich (St. Luis, MO, USA), and its average degree of polydispersity in terms of the ratio M_w_/M_n_ was 4.8. Dibutyryl chitin was synthesized using shrimp chitin. The dibutyryl chitin used in the electrospinning process had intrinsic viscosity η = 1.93 dL/g, determined using an Ubelhode viscosity meter at a temperature of 25 °C, with DMAc as solvent.

### 3.2. Types of Composites

It was proposed to produce two variant types of the composite intended for use in bone tissue regeneration, with different construction and with different values for the content of zinc alginate fibres containing TCP.

**Figure 13:**
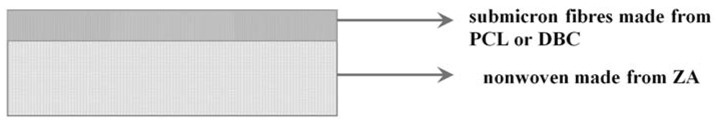
Diagram of variant I composite.

In the case of variant I the fibrous composite was made using zinc alginate fibres obtained by the traditional method of formation wet from solution. Next the fibres in segmental form were processed into nonwoven fabric using a carding device. To this fabric was applied a layer of submicron fibres made from PCL or DBC by the electrospinning method. A diagram of this model system is shown in Figure 13.

**Figure 14:**
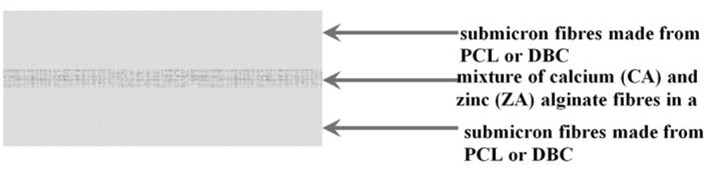
Diagram of variant II composite.

Variant II composite was produced using two layers of fibres made from PCL or DBC by the electrospinning method, between which there were introduced short fibres (2–10 mm) from a mixture of calcium and zinc alginate fibres in a ratio of 95:5. A diagram of this type of model system is shown in Figure 14.

### 3.3. Production of Alginate Fibres

**Figure 15:**
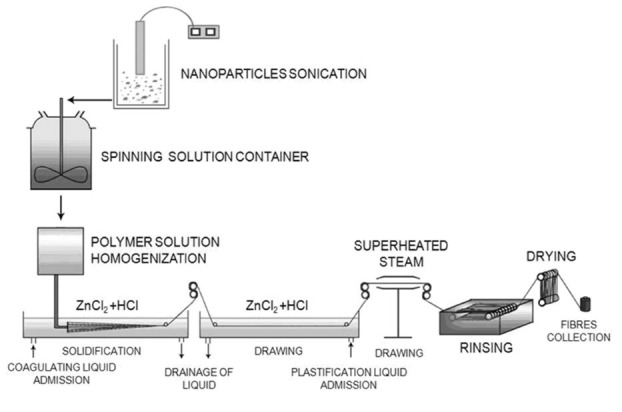
Diagram of the large laboratory spinning machine used to obtain alginate fibres [22].

Micrometric zinc or calcium alginate fibres were formed wet from solution. The fibres were produced using a large laboratory spinning machine (Figure 15). Distilled water was used as the solvent for obtaining spinning solutions. The composition and concentration of the coagulation and plasticization baths, and the parameters of the forming process (pull-out value at the spinning nozzle, total stretch) enabling the obtaining of fibres with intrinsic strength suitable for processing into nonwoven fabric, have been analysed in our previous work [19,20,21,22]. Based on the research described in [19,20,21,22], the concentration of the solution of sodium alginate in water (7.4%) and the content of nanoadditive (3% by mass of polymer) were determined. The nanoadditive (TCP), in suspension before addition to the spinning solution, was subjected to the action of ultrasound for 30 min. 

### 3.4. Production of Fabrics from DBC and PCL

**Figure 16:**
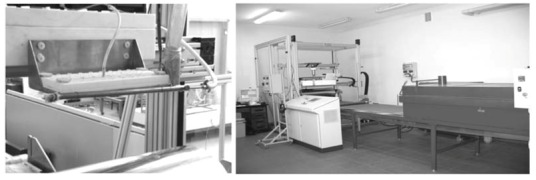
Photograph of the modified system for electrospinning.

The nonwoven fabrics made with dibutyryl chitin (DBC) and poly-ε-caprolactone (PCL) were produced by the electrospinning method using apparatus whose structure and range of operating parameters have been presented in earlier publications [26,27,28]. In the case of variant II composites, that apparatus was additionally provided with an original system for the dosing of cut alginate fibres during the electrospinning process, as shown in Figure 16.

### 3.5. Testing Methods

The *roughness of submicron fabrics* was tested using a T-500 profile tester from Hommelwerke (Villingen-Schwenningen, Germany). The measurements provided the principal roughness parameters R_a_, R_t_, R_z_. R_a_ [µm] is the arithmetic mean of the deviation of the profile. R_a_ relates to a whole elementary segment *Ir*. The representativeness of R_a_ is very limited. Single peaks have a minimal effect on the value of R_a_. R_t_ [µm] is the maximum height between the highest peak and the lowest trough. This parameter represents the vertical distance between the highest point of the filtered roughness signal within the range of the measured (computed) segment. 

R_z_ [µm] is the height of the roughness profile based on 10 points. This parameter represents the principal level of absolute values of the five highest peaks and the five lowest troughs within the range of the measured (computed) segment. Here, *Ir* is a single elementary segment on which the roughness parameters are determined.

The *wetting angle* of the nonwoven fabrics and composite materials was determined by direct measurement. The liquid used was ultra-high purity water (UHQ). Measurements were made using DSA (Drop Shape Analysis) apparatus. The results for wetting angle are means of 10 measurements (significance level α = 0.05).

The *microstructure* of nonwoven fabrics and composites was assessed based on photographs taken with the use of a scanning electron microscope (SEM JMS-5400 made by JEOL, Tokyo, Japan). 

The *absorbability* of polymeric materials was tested over 48 and 72 hours, using UHQ water as the incubation medium. Prepared water extracts (1:100) were stored at 37 °C for periods of 48 and 72 h, measuring the absorbability of the material relative to the initial sample.

*Area Density* was measured acc. EN 29073-1:1994, where the samples with surface 100 cm^2^. Samples were weighted with accuracy 0.0005 g. Obtained mass, divided by surface of sample in m^2^, gave surface density.

*Thickness of Samples* was measured acc. EN ISO 9073-2:2002 standard using thickness meter for flat nonwovens. 

*Porous Structure* of products including total pore surface and average pore size were measured using mercury porosimetry. Range of measured pore size was between 3.5 nm till 300,000 nm (pressure till 412 MPa).

In order to identify *bioactivity in vitro* test can be applied. Tested material is immersed into the simulated body fluid (SBF) [29]. Samples were immersed for 7 days in SBF in temperature 37 °C. After those 7 days the SEM + EDS analysis of the tested material have been determined due to apatite appearance in tested material.

*Biological tests* on the submicron fabrics and composites were performed using cultures of MG-63 human osteoblastoid cells. The cells were grown in MEM culture medium, with the addition of 10% foetal calf serum (PAA, Pasching, Austria) and 5% solution of antibiotics: penicillin at 10 UI/mL and streptomycin at 10 mg/mL, in an atmosphere of 5% CO_2_ and at a temperature of 37 °C. A suspension of cells was obtained by adding 5% trypsin with EDTA. After rinsing and centrifuging, the cells were brought to a concentration of 3 × 10^4^ cells per mL, and then 1 mL of the cell suspension was placed in the wells of a 24-well culture plate containing sterile discs of the tested PCL. The positive control was the polystyrene of the bottom of the wells of the culture plate (TC PS). Incubation of the MG-63 cells was carried out for 3, 7 and 14 days in an incubator (atmosphere of 5% CO_2_, temperature 37 °C). After the set time had elapsed the cells growing on the surface of the samples were subjected to tests for adhesion (CV) and viability (MTT).

*CV Cell Adhesion Test.* The ability of cells to adhere was assessed using a crystal violet absorption test. The dye absorbed by the cells was extracted, then the optical density (OD) was measured for wavelength 570 nm using an Expert Plus spectrophotometer.

*MTT Cell Viability Test.* The cytotoxicity of the material was assessed using an MTT colorimetric viability test. The activity of cells is determined using MTT dye, based on the assumption that only live cells reduce MTT to formazan crystals. In live cells MTT undergoes reduction (oxidoreduction activity of mitochondria) to purple crystals of formazan.

## 4. Conclusions

The tests carried out during this work show that alginate fibres containing TCP in a biological environment undergo a relatively rapid process of degradation. Hence this material may ensure the release of the bioactive substances added to it, in this case TCP. 

The use of large quantities of zinc alginate fibres to make composites leads to the release of too great a quantity of zinc ions, which is linked to low cell survival and a change in the pH of the culture medium. Fibres obtained by electrospinning are a biocompatible material characterized by good adhesion of cells to their surface. 

The proposed hybrid systems composed of micrometric fibres (zinc and calcium alginates containing TCP) and of submicron fibres (DBC and PCL) satisfy the requirements of regenerative medicine. The biomimetic system of fibres, the presence of a nanometric modifier, and the use of polymers with different resorption times provide a framework with specific properties on which bone cells are able to settle and proliferate. 

A biopolymer which degrades rapidly in the presence of numerous ions present in plasma (zinc alginate and calcium alginate) serves as a reservoir of bactericidal ions (Zn^2+^) as well as bioactive ones (Ca^2+^). 

## References

[1] Ratner B.D., Hoffman A.S., Schoen F.J., Lemons J.E. (2004). Biomaterials Science: An Introduction to Materials in Medicine.

[2] Ramakrishna S., Mayer J., Wintermantel E., Leong K.W. (2001). Biomedical applications of polymer-composite materials: A review. Composite Sci. Technol..

[3] Grant G.T., Morris E.R., Rees D.A., Smith P.J.C., Thom D. (1973). Biological interactions between polysacharides and divalent cations: The egg-box model. FEBS Lett..

[4] Moe S., Draget K., Skjåk-Bræk G., Smidsrød O., Stephen A.M. (1995). Alginates. Food Polysaccharides and Their Applications.

[5] Fenton J.C., Keys A.F., Mahoney P.M.J. (1998). Alginate fabric, its use in wound dressings and surgical haemostats and a process for its manufacture. U.S. Patent.

[6] Hench L.L, Jones J.R. (2005). Biomaterials, Artificial Organs and Tissue Engineering.

[7] Hashimoto T., Suzuki Y., Tanihara M., Kakimaru Y., Suzuki K. (2004). Development of alginate wound dressings linked with hybrid peptides derived from laminin and elastin. Biomaterials.

[8] Chung T.W., Yang J., Akaike T., Cho K.Y., Nah J.W., Kim S., Cho Ch.S. (2002). Preparation of alginate/galactosylated chitosan scaffold for hepatocyte attachment. Biomaterials.

[9] Dar A., Shachar M., Leor J., Cohen S. (2002). Optimization of cardiac cell seeding and distribution in 3D porous alginate scaffolds. Biotechnol. Bioeng..

[10] Draczynski Z. (2008). Honeybee corpses as an available source of chitin. J. Appl. Polym. Sci..

[11] Draczynski Z. (2008). Synthesis and solubility properties of chitin acetate/butyrate copolymers. J. Appl. Polym. Sci..

[12] Stawski D., Rabiej S., Herczyńska L., Draczynski Z. (2008). Thermogravimetric analysis of chitins of different origin. J. Therm. Anal. Cal..

[13] Krucińska I., Szosland L., Cisło R., Błasińska A., Komisarczyk A., Chilarski A., Bilska J., Pilas B. A wound dressing material of dibutyrylchityn reconstituted therefrom. Patent No.

[14] Kweon H., Yoo M.K., Park I.K., Kim T.H., Lee H.Ch., Lee H.-S., Oh J.-S., Akaike T., Cho Ch.-S. (2003). A nowel degradable polycaprolactone networks for tissue engineering. Biomaterials.

[15] Yang Q., Chen L., Shen X., Tan Z. (2006). Preparation of polycaprolactone tissue engineering scaffolds by improved solvent casting/particulate leaching method. J. Macromol. Sci. B.

[16] Kim H.-W., Knowles J.C., Kim H.-E. (2004). Hydroxyapatite/poly(e-caprolactone) composite coatings on hydroxyapatite porous bone scaffold for drug delivery. Biomaterials.

[17] Low S.W., Ng Y.J., Yeo T.T., Chou N. (2009). Use of Osteoplug polycaprolactone implants as novel burr-hole covers. Singapore Med. J..

[18] Vollath D. (2008). Nanomaterials. An Introduction to Synthesis, Properties and Applications.

[19] Boguń M., Mikołajczyk T., Rabiej S. (2009). Effect of fomation conditions on the structure and properties of nanocomposite alginate fibers. J. Appl. Polym. Sci..

[20] Mikołajczyk T., Bogun M., Kurzak A., Szparaga G. (2009). Zinc alginate fibres with a tricalcium phosphate (TCP) nanoadditive. Fibres Text. East. Eur..

[21] Boguń M., Rabiej S. (2010). The influence of fiber formation conditions on the structure and properties of nanocomposite alginate fibers containing tricalcium phosphate or montmorillonite. Polym. Compos..

[22] Boguń M. (2010). Nanokompozytowe włókna alginianowe i kompozyty z ich udziałem do zastosowań w inżynierii biomateriałowej.

[23] Bogun M., Stodolak E., Mikolajczyk T., Krucinska I., Błazewicz M. Biodegradable Composites Based on the Alginate Fibres and Polycaprolactone, and Dibutyrylchitin for Applications in Regeneration of the Osseous Tissue. Proceedings of FIBREMED 2011.

[24] Beachleya V., Wen X. (2010). Polymer nanofibrous structures: Fabrication, Biofunctionalization, and cell interactions. Prog. Polym. Sci..

[25] Xiang P., Li M., Zhang Ch.-Y., Chen D.-L., Zho Z.-H. (2011). Cytocompatibility of electrospun nanofiber tubular scaffolds for small diameter tissue engineering blood vessels. Inter. J. Biol. Macrom..

[26] Krucinska I., Gliscinska E., Chrzanowski M. System for electrospinning fibres. Patent No.

[27] Krucinska I., Komisarczyk A., Chrzanowski M., Gliscinska E., Wrzosek H. (2009). Electrostatic field in electrospinning with a multicapillary head-modeling and experiment. Fibres Text. East. Eur..

[28] Krucinska I., Gliscinska E., Chrzanowski M., Komisarczyk A. Multi-nozzle Laboratory Stand for Electrospinning Process. Proceedings of 10th World Textile Conference, AUTEX 2010.

[29] Kokubo T., Takadama H. (2006). How useful is SBF in predicting *in vivo* bone activity. Biomaterials.

